# Comparative Regenerative Efficacy of PRP Combined with Chondrocytes or Mesenchymal Stem Cells for Intervertebral Disc Regeneration in a Rabbit Model

**DOI:** 10.3390/ijms262010007

**Published:** 2025-10-14

**Authors:** Pedro M. Reyes-Fernandez, Viktor J. Romero-Díaz, Jaime García Juárez, José F. Vílchez-Cavazos, Carlos A. Acosta-Olivo, Víctor M. Peña-Martínez, Jorge Lara-Arias

**Affiliations:** 1 Orthopedics and Traumatology Service, “Dr. José Eleuterio González” University Hospital, Universidad Autónoma de Nuevo León, Monterrey 66460, Mexico; pedro.reyesfr@uanl.edu.mx (P.M.R.-F.); jose.vilchezcvz@uanl.edu.mx (J.F.V.-C.); carlos.acostalv@uanl.edu.mx (C.A.A.-O.); 2 Histology Department, Faculty of Medicine, Universidad Autónoma de Nuevo León, Monterrey 66460, Mexico; vikromero@email.com (V.J.R.-D.); jaime.garciajr@uanl.edu.mx (J.G.J.)

**Keywords:** intervertebral disc degeneration, PRP, chondrocytes, mesenchymal stem cells, disc regeneration, animal model

## Abstract

Intervertebral disc degeneration is a leading cause of chronic back pain, with existing treatments focusing on symptom management rather than true tissue repair. Cellular therapies—such as platelet-rich plasma (PRP), autologous chondrocytes, and mesenchymal stem cells (MSCs)—have emerged as promising strategies for disc regeneration. In this study, fifteen New Zealand white rabbits underwent fluoroscopy-guided needle puncture of the L4-L5 discs and were allocated to receive PRP alone, PRP-chondrocytes, or PRP-MSCs eight weeks later, while the L3-L4 disc served as a healthy internal control. At 16 weeks post-injury, histological scoring revealed significant improvements in annular integrity, cellularity, and matrix composition in all treated groups compared with untreated lesions, with the greatest gains observed in the PRP-chondrocytes arm, intermediate effects with PRP-MSCs, and more modest changes with PRP alone. Complementary RT-qPCR analysis of COL2A1 and COL10A1 expression confirmed a shift toward a more regenerative phenotype, marked by enhanced COL2A1 and reduced COL10A1 levels, which was most pronounced in the PRP-chondrocytes arm. Despite these advances, none of the interventions fully restored the healthy disc architecture, underscoring the complexity of disc repair. These findings support the potential of combining PRP with chondrocytes or MSCs for intervertebral disc regeneration and demonstrate the need for further optimization of cell doses, PRP formulations, and delivery protocols before clinical translation.

## 1. Introduction

The intervertebral disc is a fibrocartilaginous structure composed of the nucleus pulposus, surrounded by the annulus fibrosus and enclosed by the vertebral endplates. The extracellular matrix (ECM) of the intervertebral disc plays a crucial role in biomechanical functioning and cellular homeostasis. It is primarily composed of a dense collagen network, proteoglycans, and water, along with non-collagenous proteins, glycoproteins, and elastin, which provide structural integrity and facilitate cellular signaling. It acts as a shock absorber by transmitting compressive loads between the vertebral bodies and enables the various movements of the spine through a complex joint system. Among all of the musculoskeletal tissues, the intervertebral disc is considered to undergo degenerative changes at a younger age [1,2,3]. With degeneration and aging, histomorphological and functional changes occur, leading to decreased permeability of the endplates and reduced vascular supply, which, in turn, impair metabolite transport. Proteoglycans begin to fragment and their overall content decreases with age, particularly in the nucleus pulposus. In parallel, there is an increase in collagen content, whereby type I collagen fibers in the inner annulus fibrosus and nucleus pulposus are replaced by type II collagen fibers. In addition, ECM turnover decreases in the aged disc, allowing collagen fibers to become more reticular, which leads to the retention of damaged fibers and reduced tissue strength [4].

At present, the gold standard for treating disc degeneration and the mechanical consequences of disc structural loss—such as discogenic pain, axial instability, translational and angular instability, disc herniations, or narrowing of the spinal canal—is spinal fusion with or without fixation and/or laminectomy. This approach follows the principle of “no disc, no pain.” Other treatments aimed at preserving segmental movement, such as disc prostheses or dynamic devices, have also been developed [5]. Additionally, minimally invasive techniques have been introduced to facilitate faster recovery and prevent delayed dural complications. However, these techniques are only moderately successful, with relatively high reoperation rates. This is primarily due to the replacement of the natural disc structure with fibrous scar tissue, which often leads to further complications [6].

Given the high costs associated with this condition and the discomfort it causes, new treatment modalities focusing on cellular approaches that restore internal homeostasis and recover the extracellular matrix have been explored [7]. One such approach involves mesenchymal stem cells (MSCs), which can be found in bone marrow and various adult tissues. These cells possess pluripotent properties and the ability to replicate and differentiate into other mesenchymal tissues, including bone, cartilage, fat, tendons, and muscle [8,9]. In the context of the intervertebral disc, MSCs have been shown to halt the progression of disc degeneration and promote an increase in disc height, offering a potential treatment option for degenerative disc disease [10]. Another approach involves autologous articular chondrocytes, which serve as a viable source due to their phenotypic similarity to disc cells and the fact that they can be harvested from non-load-bearing areas of the knee. Articular defects have been successfully repaired using autologous chondrocytes, suggesting that these cells can survive transplantation and contribute to tissue repair [11]. This technique, involving the implantation of living cells through chondrocyte cultures, appears to be a promising approach for inducing the restoration of the intervertebral disc’s functional extracellular matrix [12]. Furthermore, platelet-rich plasma (PRP) contains a high concentration of growth factors and cytokines, which can promote the migration and proliferation of various cells, thus fostering a favorable microenvironment for the repair and regeneration of musculoskeletal tissues. In vitro studies have demonstrated that PRP effectively promotes cell proliferation and extracellular matrix metabolism in intervertebral discs in animal models [13].

As PRP contains chondrogenic growth factors, it may stimulate extracellular matrix synthesis when administered alone, while, in combination with MSCs, it may enhance their differentiation toward a chondrocytic phenotype. However, since chondrocytes do not differentiate, they will use their own chondrogenic factors for extracellular matrix synthesis when applied. This led us to assume that the latter treatment will be more effective than PRP or MSCs/PRP when infiltrated into damaged intervertebral discs, such as those developed in the animal model described by Masuda et al. in 2005 [14].

The objective of this study is to analyze the therapeutic effect of PRP—administered alone or in combination with chondrocytes or MSCs—in an animal model of intervertebral disc injury. The working hypothesis is that the infiltration of chondrocytes combined with PRP will stimulate better tissue regeneration than treatments based solely on PRP or MSCs/PRP in such a model.

## 2. Results

### 2.1. Group A: PRP Treatments

The L2-L3 discs were injured through fluoroscopy-guided puncture and subjected to a degenerative process, then evaluated using a histological classification scale. Regarding the annulus fibrosus grade, three discs exhibited pattern 2, with broken or wavy fibers in less than 30% of the annulus, while two discs showed pattern 3, with more than 30% of the annulus affected. Concerning the boundary between the annulus fibrosus and the nucleus pulposus, three discs were classified as grade 2, indicating minimal disruption, and two as grade 3, indicating moderate-to-severe disruption.

Cellularity was assessed using a qualitative histological grading scale, rather than direct cell counting. The evaluation was conducted following the methodology described by Masuda et al. [14], which provides a standardized scoring system for intervertebral disc degeneration. Each histological section was analyzed in three randomly selected high-power fields (40× magnification), ensuring representation of both the central and peripheral regions of the nucleus pulposus and annulus fibrosus. The scores were assigned based on established histological grading criteria to ensure consistency and reproducibility.

For nucleus pulposus cellularity, two discs were classified as grade 2, indicating a slight reduction in cell number and fewer vacuoles, while three discs were classified as grade 3, showing a moderate-to-severe (50%) reduction in cell number and absence of vacuoles. Regarding the nucleus pulposus matrix, two discs received a grade 2 classification, corresponding to slight condensation of the extracellular matrix, while three discs were classified as grade 3, indicating moderate-to-severe matrix condensation. The intervention resulted in significant degeneration in the L2-L3 discs of Group A, with total scores ranging from 8 to 12, reflecting moderate-to-severe degeneration.

The L3-L4 discs, maintained as unpunctured healthy controls, preserved normal characteristics in all specimens according to the histological scale, with most discs achieving a score of 4, corresponding to a normal state.

Finally, for the L4-L5 discs—which were injured and subsequently treated with PRP—variability was observed in tissue recovery. Regarding the annulus fibrosus grade, all five discs exhibited pattern 1, corresponding to the normal fibrocartilage lamellae pattern (U-shaped posteriorly and slightly convex anteriorly), with no broken or wavy fibers. In terms of the boundary between the annulus fibrosus and the nucleus pulposus, one disc was classified as grade 1, indicating a normal state, while four discs showed grade 2, corresponding to minimal disruption.

**Figure 1:**
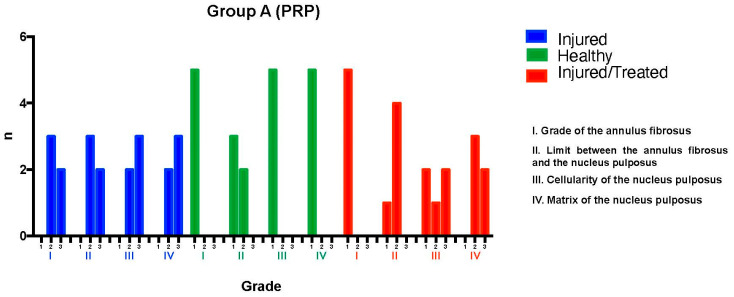
Group A. L2-L3 (I) exhibited moderate-to-severe degeneration, with scores ranging from 8 to 12 points. L3-L4 (H) maintained normal characteristics, consistently scoring 4 points, indicating a completely normal state. L4-L5 (I-T) showed improvement compared to the injured but untreated disc (L/no T) but did not fully reach the normal characteristics of disc tissue, with scores ranging from 6 to 7 points.

For nucleus pulposus cellularity, two discs were classified as grade 1, indicating normal cellularity with large vacuoles in the gelatinous matrix structure. One disc was classified as grade 2, indicating minimal disruption, and two discs were classified as grade 3, showing a moderate-to-severe (50%) reduction in cell number and absence of vacuoles. Regarding the nucleus pulposus matrix, three discs were classified as grade 2, indicating slight condensation of the extracellular matrix, while two were classified as grade 3, corresponding to moderate-to-severe extracellular matrix condensation. The overall histological scores ranged from 6 to 7, indicating improvement compared to untreated injured discs but falling short of restoring normal disc tissue. Although the intervention showed a tendency to preserve certain disc characteristics despite the degenerative process, its regenerative effect was limited (Figure 1).

**Figure 2:**
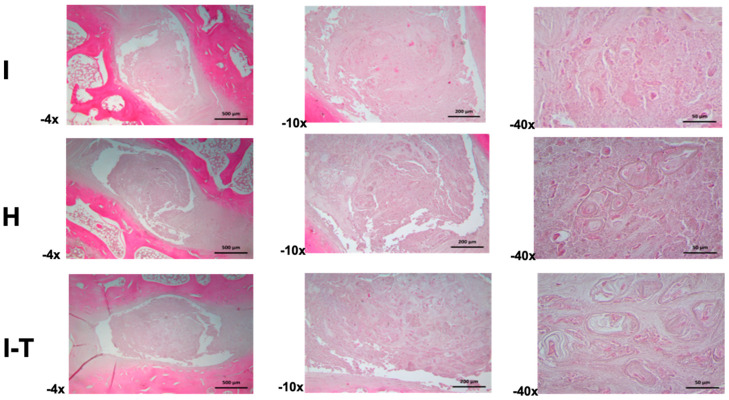
Histological comparison of intervertebral discs in Group A treated with PRP. From left to right and top to bottom: I, Injured L2-L3 discs (4× and 10×), where the annulus fibrosus exhibits variable thickness and the nucleus pulposus presents a mixed extracellular matrix; at 40×, cellular irregularities are highlighted, with some chondroid phenotypes and others undergoing atrophic or necrotic processes. H, Healthy L3-L4 discs (4× and 10×), showing well-aligned collagen bundles in the annulus fibrosus and an amorphous matrix in the nucleus pulposus, with irregularly sized cells; at 40×, hypertrophic cartilaginous lacunae with hyperchromatic cells are observed, surrounded by finely granular material. I-T, Injured and PRP-treated L4-L5 discs (4× and 10×), which present a normal annulus fibrosus structure and a nucleus pulposus rich in cellular nests; at 40×, these cellular structures are surrounded by a heterogeneous extracellular matrix. (I: injured; H: healthy; I-T: injured and treated) H&E staining.

Regarding the microscopic findings for Group A (Figure 2) in the injured L2-L3 disc (4× and 10×), the annulus fibrosus exhibited variable thickness and was composed of regularly organized collagen bundles. The nucleus pulposus was characterized by a mixed extracellular matrix, with scattered small- to medium-sized cells. At higher magnification (40×), morphological irregularities were observed in the cells, with some displaying a chondroid phenotype and others showing signs of atrophy or necrotic processes, all embedded in a granular extracellular matrix.

For the healthy L3-L4 disc (4× and 10×), the annulus fibrosus showed parallel-aligned collagen bundles with sparse cellular inclusions. The nucleus pulposus consisted of an amorphous and fibrous matrix housing irregularly sized cells, sometimes isolated or grouped, and occasionally forming cellular nests. At higher magnification (40×), some hypertrophic cartilaginous lacunae were identified, containing small, hyperchromatic cells with signs of atrophy or necrosis, surrounded by finely granular material and cellular debris.

For the injured and PRP-treated L4-L5 disc (4× and 10×), the annulus fibrosus revealed variable thickness with a normal structure. The nucleus pulposus comprised abundant cellular nests of various shapes and sizes, embedded in an amorphous extracellular matrix. At higher magnification (40×), the cellular nests included hypertrophic lacunae; some exhibited atrophic or necrotic cells. The extracellular matrix appeared heterogeneous, with amorphous regions and finely granular areas.

### 2.2. Group B: PRP-Chondrocyte Treatments

After fluoroscopy-guided puncture and the development of the degenerative process, the injured L2-L3 discs were analyzed and evaluated using the histological grading scale. Regarding the annulus fibrosus grade, one disc exhibited mild damage, with less than 30% of the annulus presenting broken or wavy fibers (pattern 2); meanwhile, the damage was more extensive in four discs, affecting more than 30% of the annulus (pattern 3). Concerning the boundary between the annulus fibrosus and the nucleus pulposus, two discs showed minimal disruption (grade 2), while three exhibited moderate-to-severe disruption (grade 3), indicating more significant deterioration in most cases.

Regarding nucleus pulposus cellularity, three discs showed a slight reduction in the number of cells and fewer vacuoles (grade 2), while the reduction was moderate-to-severe in two discs, with a 50% decrease in the number of cells and absence of vacuoles (grade 3), suggesting a significant loss of cellular viability in these cases. Finally, all discs exhibited moderate-to-severe condensation of the nucleus pulposus extracellular matrix (grade 3), reflecting an advanced stage of tissue degeneration throughout the evaluated sample. These results indicate a significant degree of degeneration in the examined intervertebral discs, with variations in the extent of structural and cellular damage observed.

The intervention in the Group B L2-L3 discs resulted in severe degeneration, as evidenced by the consistently high scores reflecting substantial deterioration. Of the five evaluated discs, four reached a total of 11 points, distributed as follows: two of these discs received 3 points in categories I, II, and IV and 2 points in category III, while the other two discs obtained 3 points in categories I, III, and IV and 2 points in category II. The fifth disc received a total of 10 points, with 3 points assigned to categories II and IV and 2 points to categories I and III. These findings indicate severe degeneration in the treated discs within this group.

The L3-L4 discs, maintained as unpunctured healthy controls, preserved normal characteristics according to the histological scale. All discs showed pattern 1 for the annulus fibrosus grade, corresponding to normal fibrocartilage lamellae with no broken or wavy fibers. In the boundary between the annulus fibrosus and the nucleus pulposus, one disc was graded as 1 (normal) and four discs as 2 (minimally disrupted). Nucleus pulposus cellularity was normal (grade 1) in four discs, with large vacuoles, and slightly reduced (grade 2) in one disc. The nucleus pulposus matrix was classified as grade 1 (normal gelatinous appearance) in all discs. These results confirmed that the L3-L4 control discs maintained their normal state, with overall scores consistently ranging from 4 to 6 points.

**Figure 3:**
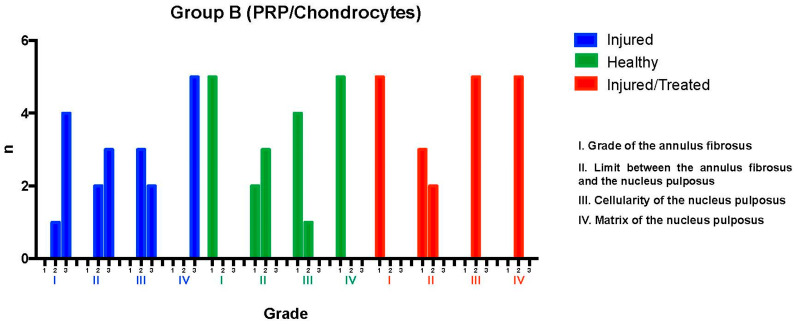
Group B. L2-L3 (I) exhibited severe degeneration, with scores of 11 points in 4 samples and 10 points in 1 sample. L3-L4 (H) maintained normal characteristics, with a consistent overall score ranging from 4 to 6 points. L4-L5 (I-T) showed improvement compared to the injured but untreated disc (L/no T) but did not fully achieve the normal characteristics of disc tissue, with scores ranging from 6 to 7 points.

Finally, in the L4-L5 injured and PRP-chondrocyte-treated discs, all discs exhibited pattern 1 for the annulus fibrosus grade, indicative of a normal state. For the boundary between the annulus fibrosus and the nucleus pulposus, three discs were graded as 1 (normal), and two as 2 (minimally disrupted). Nucleus pulposus cellularity showed minimal disruption (grade 2) in all five discs, and the nucleus pulposus matrix was graded as 2 (slight condensation) in all discs. This evaluation revealed that the L4-L5 discs, following treatment with PRP-chondrocytes, exhibited variability in their ability to preserve normal characteristics against degenerative damage; in particular, their scores ranged from 6 to 7 points, reflecting a trend toward regeneration of previously injured tissue (Figure 3).

**Figure 4:**
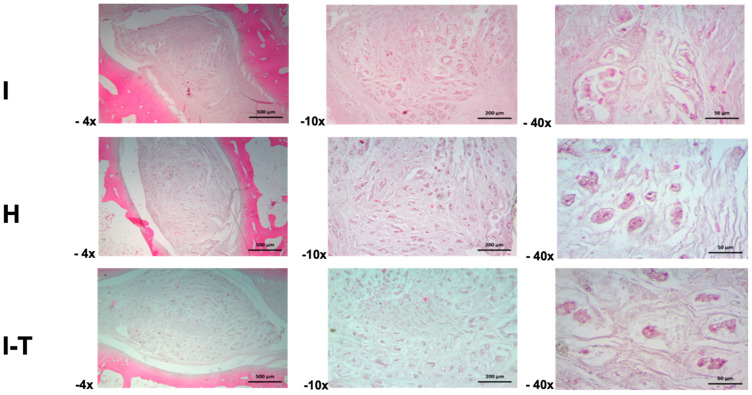
Histological evaluation of intervertebral discs in Group B. The images capture the tissue architecture at different magnifications (4×, 10×, and 40×), highlighting the diversity in cellular morphology and structure across injured (I), healthy (H), and PRP-chondrocyte-treated discs (I-T). Variations in the integrity of the annulus fibrosus, irregularities in cartilaginous lacunae, and alterations in the extracellular matrix are observed, demonstrating the combined effects of the injury and subsequent treatment. (I: injured; H: healthy; I-T: injured and treated) H&E staining.

The microscopic findings for Group B (Figure 4) indicate that, in the injured L2-L3 disc, the intervertebral disc exhibited matrix retraction changes at 4× magnification, resulting in irregular spaces and atrophic cartilaginous lacunae. At 10×, the extracellular matrix of the nucleus pulposus revealed atrophic pseudocartilaginous lacunar structures surrounded by a fibrillar matrix. At 40×, detailed structural aspects of the nucleus pulposus and retracted cellular lacunae embedded in a mixed amorphous and fibrillar matrix were observed.

In the healthy L3-L4 disc, an irregular matrix was identified at 4× magnification, with groups of cellular lacunae surrounded by a heterogeneous matrix, showing nucleus pulposus retraction that creates spaces with the annulus fibrosus. At 10×, irregularly shaped cartilaginous lacunae surrounded by a finely fibrillar matrix were observed. At 40×, the cartilaginous lacunae displayed altered cell morphology due to atrophy, with basophilic cytoplasmic staining, all embedded in a heterogeneous fibrillar matrix.

The injured and PRP-chondrocyte-treated L4-L5 disc showed separation of the nucleus pulposus from the annulus fibrosus at 4× magnification, with the latter exhibiting contraction of fibrous fascicles. The nucleus pulposus presented a mixed amorphous and fibrillar matrix with scattered cellular lacunae. At 10×, the cartilaginous matrix of the nucleus pulposus included lacunae with an atrophic appearance, creating spaces within the matrix components. At 40×, details of the nucleus pulposus matrix revealed atrophic cartilaginous lacunae surrounded by a heterogeneous and fibrillar matrix.

### 2.3. Group C: PRP-MSCs

The L2-L3 discs were injured through fluoroscopy-guided puncture and subjected to a degenerative process, then evaluated using the histological classification scale. Regarding the grade of the annulus fibrosus, three discs predominantly exhibited pattern 2, with broken or wavy fibers in less than 30% of the annulus, while two discs showed pattern 3, with more than 30% of the annulus affected. In terms of the boundary between the annulus fibrosus and the nucleus pulposus, three discs were graded as 2, indicating minimal disruption, and two discs were graded as 3, indicating moderate-to-severe disruption. Regarding the nucleus pulposus cellularity, all five discs were classified as grade 3, showing a moderate-to-severe (50%) reduction in cell number and absence of vacuoles. In relation to the nucleus pulposus matrix, all discs received a grade of 3, indicating moderate-to-severe extracellular matrix condensation. It was concluded that the intervention led to significant degeneration in the Group C L2-L3 discs, with consistent results: two discs scored 12 points, with three points in categories I, II, III, and IV; and three discs scored 10 points, with two points in categories I and II and three points in categories III and IV, indicating severe degeneration.

The L3-L4 disc, maintained as an unpunctured healthy control, was evaluated using the same scale. All five discs showed pattern 1 in the annulus fibrosus grade, indicative of a normal state with no broken or wavy fibers. In the boundary between the annulus fibrosus and the nucleus pulposus, two discs were graded as 1, corresponding to a normal state, and three discs were graded as 2, indicating minimal disruption. Nucleus pulposus cellularity was normal—i.e., grade 1—in three discs, with large vacuoles, and slightly reduced—i.e., grade 2—in two discs. All discs exhibited a normal nucleus pulposus matrix, classified as grade 1. This demonstrated that the L3-L4 discs maintained their normal characteristics, with scores ranging between 4 and 6 points, indicating the preservation of normal conditions in most cases.

**Figure 5:**
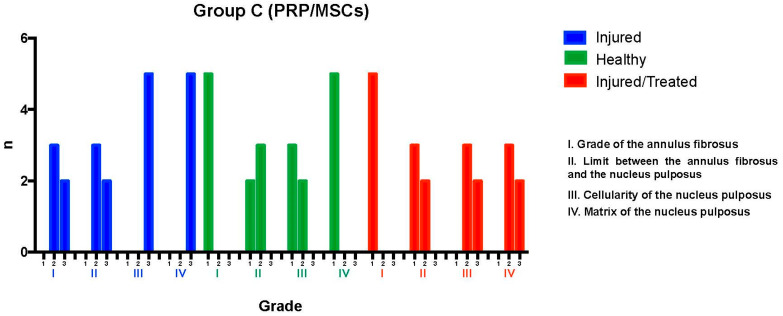
L2-L3 (I) exhibited severe degeneration, with scores of 12 points in 2 samples and 10 points in 3 samples. L3-L4 (H) maintained normal characteristics, with a consistent general score ranging from 4 to 6 points. L4-L5 (I-T) showed improvement compared to the injured but untreated disc (L/no T) but did not fully reach the normal characteristics of disc tissue, with scores ranging from 6 to 8 points.

Finally, the L4-L5 discs, which had been injured and subsequently treated with PRP-MSCs, were analyzed. All discs exhibited pattern 1 for the annulus fibrosus grade, reflecting a normal state. Regarding the boundary between the annulus fibrosus and the nucleus pulposus, three discs were graded as 1 (normal) and two discs as grade 2 (minimally interrupted). Nucleus pulposus cellularity was classified as grade 2 in all discs, indicating minimal disruption. The nucleus pulposus matrix was graded as 2 in three discs and as 3 in two discs, indicating mild to moderate condensation. This evaluation determined that the L4-L5 discs exhibited variable characteristics after treatment with PRP-MSCs, suggesting partial preservation of normal features against degenerative damage, with consistent scores ranging from 6 to 8 points reflecting a modest regenerative effect (Figure 5).

**Figure 6:**
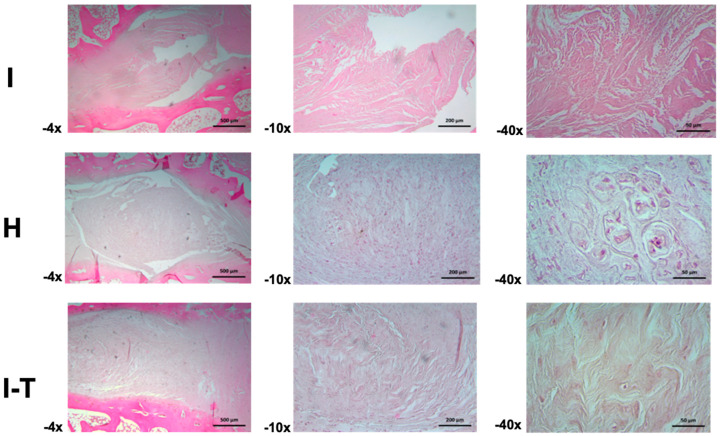
Histological analysis of intervertebral discs in Group C treated with PRP and MSCs. The images depict the cellular composition and matrix architecture in injured (I), healthy (H), and injured-treated discs (I-T) (4×, 10×, and 40×). The organization of fibrocollagenous bundles, the density and morphology of cells in the nucleus pulposus, and the differences in the extracellular matrix following treatment are observed. These findings provide detailed insights into tissue alterations and the response to treatment in the intervertebral disc. (I: injured; H: healthy; I-T: injured and treated) H&E staining.

Regarding the microscopic analysis for Group C (Figure 6) at 4× and 10× magnifications, the injured L2-L3 disc was observed to be composed of fibrocollagenous bundles organized both longitudinally and perpendicularly, with visible artifacts. At 40×, areas of the fibrous matrix displayed a granular appearance, suggesting possible fragmentation of fibrous fascicles or transverse sections of the same.

In the healthy L3-L4 disc, the annulus fibrosus appeared narrow at 4× magnification, with limited parallel fiber bundles, while the nucleus pulposus was dense and highly cellular, with a predominantly fibrous but dispersed and irregular matrix and basophilic cells. At 10×, cells appeared ovoid and stellate in the central region of the nucleus pulposus, with some fusiform and basophilic cells. Toward the periphery, rounded cell clusters formed nests separated by scant amorphous and fibrous matrix. At 40×, details of chondroid-like cellular nests with well-defined capsules and wide niches were observed.

For the injured and PRP-MSC-treated L4-L5 disc, the annulus fibrosus showed a dense cellular population among the fibers at 4× magnification, extending into the nucleus pulposus and forming thicker and more cellular bundles. At 10×, ovoid, fusiform, and rounded cells dispersed among the fibers were distinguishable. At 40×, the fibrocollagenous bundles appeared perpendicular, with sparse amorphous ground substance and chondroid cells embedded in their respective lacunae.

### 2.4. Statistical Results

In this study, a sample of 15 rabbits was used, which were distributed into three groups of 5 rabbits each. Each rabbit underwent an induced injury in two intervertebral discs, followed by different treatments according to their group: the first group received platelet-rich plasma (PRP), the second group was treated with PRP and chondrocytes, and the third group was treated with PRP and mesenchymal stem cells (MSCs). After treatment administration, the rabbits were euthanized, and a histological evaluation was conducted on the two injured intervertebral discs, as well as on an adjacent healthy intervertebral disc.

The results presented in Table 1 allow for a comparison of the scores obtained in the intervertebral discs evaluated among the three treatment groups. In the healthy intervertebral discs, the average histological scores ranged between 4.4 and 5 across all three groups, with no statistically significant differences observed (*p* = 0.42). In the injured but untreated discs, the average values ranged from 10 to 10.8 among the groups (*p* = 0.46). In contrast, for the injured and treated discs—whether treated with PRP alone, PRP with chondrocytes, or PRP with MSCs—the average values ranged between 6.4 and 7.2 (*p* = 0.11).

When analyzing the differences among the intervertebral discs (healthy, injured, and injured with treatment) within each group, statistically significant differences were found (*p* < 0.001). The average scores for histological integrity were 4.73 ± 0.70 for healthy discs, 10.53 ± 1.12 for injured discs, and 6.93 ± 0.70 for injured and treated discs.

In the comparison between groups, significant differences were observed among all conditions. The scores for the healthy discs differed from those for both the injured untreated discs and the injured treated discs. Likewise, the injured untreated discs differed from the injured treated discs, with the latter failing to reach the values of the healthy discs. Significant differences in the histological integrity of treated and untreated intervertebral discs across the three treatment groups were demonstrated, suggesting the beneficial effects of treatment with PRP, chondrocytes, and MSCs compared to untreated injured discs.

The healthy discs across all three groups maintained consistent histological scores and showed no significant differences—this was expected, as they were not subjected to intervention. These data serve as a baseline, reflecting disc integrity under normal conditions. In the untreated injured discs, the scores were also consistent and showed no significant differences between groups, establishing the homogeneity of the induced disc damage prior to treatment. This is a crucial point for validating the comparability of post-treatment results.

The variability in scores among the treated discs indicates that the treatments had a differential effect. The treated injured discs in groups treated with PRP and PRP-chondrocytes showed a trend toward better scores compared to the untreated injured discs, suggesting beneficial effects on the histological integrity of the disc. However, it is noteworthy that, although improvements were observed, none of the treatments fully restored the histological score to that of a healthy disc.

### 2.5. Molecular Analysis

**Figure 7:**
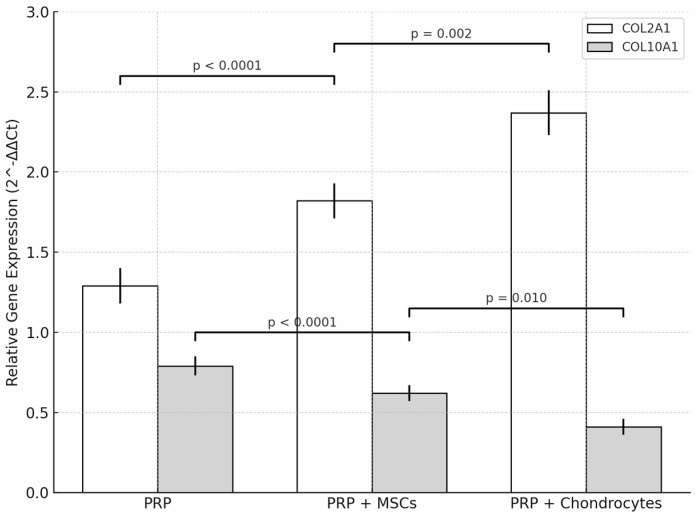
Relative expression of COL2A1 and COL10A1 mRNA in intervertebral discs treated with PRP, PRP + mesenchymal stem cells (MSCs), or PRP + chondrocytes. Data represent mean ± SD (n = 5). Expression levels were normalized to *β*-actin and calibrated to lesion controls (L2-L3) using the 2^−ΔΔCt method. The PRP + chondrocyte treatment yielded the highest COL2A1 expression and the lowest COL10A1 expression among groups. One-way ANOVA followed by Tukey’s post hoc test was performed for statistical analysis. *p*-values are shown for significant intergroup comparisons.

Quantitative RT-qPCR demonstrated that all treatments altered COL2A1 and COL10A1 expression relative to lesion controls (L2-L3). In the PRP-chondrocytes group, COL2A1 in treated discs (L4-L5) increased from 1.03 ± 0.12 to 2.37 ± 0.14 (*p* = 0.002), corresponding to a 2.3-fold elevation, while COL10A1 declined from 1.05 ± 0.06 to 0.41 ± 0.05 (*p* = 0.003), a 0.4-fold level compared to lesion controls (Figure 7).

Relative to healthy controls (L3-L4; COL2A1 = 3.08 ± 0.17; COL10A1 = 0.22 ± 0.04), PRP-chondrocytes-treated discs reached 2.37 ± 0.14 for COL2A1 (0.77-fold of healthy; *p* = 0.010) and 0.41 ± 0.05 for COL10A1 (1.9-fold of healthy; *p* = 0.030).

The PRP-MSCs protocol induced COL2A1 expression at a level of 1.82 ± 0.11 in treated discs, for a 1.9-fold increase over lesion controls (0.98 ± 0.11; *p* = 0.010), and reduced COL10A1 to 0.62 ± 0.05 (0.61-fold vs. lesion controls at 1.02 ± 0.05; *p* = 0.015). Compared to healthy discs, COLA1 in this group was 0.62-fold (1.82 ± 0.11 vs. 2.95 ± 0.16; *p* = 0.005), while COL10A1 was 2.9-fold (0.62 ± 0.05 vs. 0.19 ± 0.03; *p* = 0.008).

PRP alone produced a modest elevation in COL2A1 to 1.29 ± 0.11 (1.3-fold vs. lesion controls at 1.01 ± 0.10; *p* = 0.080) and decreased COL10A1 to 0.79 ± 0.06 (0.80-fold vs. lesion controls at 0.99 ± 0.05; *p* = 0.040). Compared to healthy discs, COL2A1 was 0.43-fold (1.29 ± 0.11 vs. 3.02 ± 0.14; *p* < 0.001), while COL10A1 was 4.4-fold (0.79 ± 0.06 vs. 0.18 ± 0.04; *p* < 0.001).

Intergroup comparison of treated discs confirmed significant differences in marker expression. For COL2A1, the mean values were 2.37 ± 0.14 (PRP-chondrocytes), 1.82 ± 0.11 (PRP-MSCs), and 1.29 ± 0.11 (PRP) (one-way ANOVA, F (2.12) = 56.8, *p* < 0.0001; Tukey’s post hoc: PRP-chondrocytes > PRP-MSCs, *p* = 0.002; PRP-MSCs > PRP, *p* < 0.0001). For COL10A1, the mean values were 0.41 ± 0.05, 0.62 ± 0.05, and 0.79 ± 0.06, respectively (one-way ANOVA, F (2.12) = 28.3, *p* < 0.0001; Tukey’s post hoc: PRP-chondrocytes < PRP-MSCs, *p* = 0.010; PRP-MSCs < PRP, *p* < 0.0001). These results indicate that PRP combined with chondrocytes yielded the most favorable chondrogenic profile and the lowest hypertrophic marker expression, in agreement with the histological findings.

## 3. Discussion

This study evaluated the reparative efficacy of three biologically based strategies—PRP alone, PRP combined with chondrocytes, and PRP combined with mesenchymal stem cells—in intervertebral disc (IVD) regeneration in a rabbit model of disc injury. Histological readouts were complemented with targeted molecular profiling via quantitative RT-qPCR of key chondrogenic and hypertrophic markers (COL2A1 and COL10A1), providing a mechanistic context to the tissue-level observations.

Histologically, PRP produced variable improvements among the treated discs (L4-L5) relative to injured controls (L2-L3), without fully restoring the structural and cellular integrity of the nucleus pulposus and annulus fibrosus. These findings are consistent with prior work showing that, while PRP can stimulate cell proliferation and extracellular matrix synthesis, the outcome of treatment depends on injury severity, formulation, and delivery parameters [15,16]. Sources of variability likely include heterogeneity in PRP composition, baseline lesion severity, and inter-individual biological responses [17,18]. At the molecular level, PRP induced only partial changes, consistent with an earlier annular puncture study in which autologous PRP increased COL2A1 without a concomitant rise in COL10A1, suggesting a modest chondrogenic effect without promoting hypertrophy [19]. Collectively, PRP alone confers partial histological and molecular benefits, but may be insufficient for complete IVD regeneration in this model [15,16,17,18,19].

PRP combined with chondrocytes resulted in more consistent structural repair, including normalization of fibrocartilage lamellae, in line with the reparative potential of cartilage-derived cells. The existing literature supports the rationale for combining PRP with chondrocytes to restore cartilage-like ECM [20,21,22,23] and highlights potential synergy in PRP cell constructs for cartilage repair [24,25], aligning with broader strategies to address causal drivers of IVD degeneration beyond symptomatic relief [26]. Our molecular analysis confirmed these improvements: COL2A1 expression increased, while COL10A1 decreased toward levels seen in healthy discs, echoing evidence from canine models of disc-chondrocyte transplantation where cells supported type II collagen synthesis without excessive hypertrophy [27]. Together, these data and prior reports support PRP-chondrocyte therapy as a promising strategy for disc repair [20,21,22,23,24,25,26,27].

Similarly, PRP combined with MSCs enhanced disc repair when compared with injured controls, consistent with prior ex vivo and in vivo studies showing that PRP potentiates MSC chondrogenesis and matrix deposition in disc-like environments [28]. The degree of regeneration appears to be context-dependent, influenced by the severity of injury, MSC phenotype, and microenvironmental cues [29]. At the molecular level, discs treated with PRP-MSCs demonstrated improved COL2A1/COL10A1 balance relative to injury, consistent with antidegenerative effects previously reported in rabbit models [30] and in the broader MSC literature [28,29].

Overall, both combination strategies outperformed PRP alone across histological and molecular outcomes, supporting a synergistic interaction between PRP and reparative cells [20,21,22,23,24,25,26,28,29]. Our integrated findings argue for combination approaches that couple optimized PRP formulations with cellular therapies. While previous studies have explored the use of chondrocytes or MSCs alone for disc regeneration [27,28,29,30], our experimental design did not include groups with cells administered without PRP. This was a deliberate choice, as the central hypothesis of the present study was to evaluate the potential synergistic effects of combining PRP with reparative cells. We recognize that this limits direct comparisons with cell-only therapies, which should be addressed in future investigations. Nevertheless, based on our findings and prior evidence, the combined strategies may offer distinct advantages over PRP alone by enhancing extracellular matrix synthesis, sustaining a chondrogenic phenotype, and reducing hypertrophic drift, thereby potentially improving clinical outcomes in patients with disc degeneration.

Combined approaches may offer clinical advantages over PRP alone in patients with disc degeneration. While PRP provides a rapid but transient release of growth factors, its effects may wane as the degenerative environment persists. In contrast, adjunct chondrocytes or MSCs can sustain matrix synthesis and exert paracrine immunomodulatory activity, helping to maintain a chondrogenic phenotype and reduce hypertrophic drift. These mechanisms may prolong the therapeutic benefit beyond that achieved by PRP alone. Clinically, such combined strategies could provide more durable symptom relief and structural repair, representing a potential step forward when compared with PRP-only interventions.

Inter- and intra-study variability likely reflects differences in PRP composition (platelet/leukocyte content, activation), injury severity, cell phenotype and dose, and delivery kinetics [17,18,29]. Going forward, approaches for the standardization of PRP preparation, rigorous cell characterization, and tuning of carrier mechanics may reduce variance and improve effect sizes [15,16,20,21,24,25,26]. Building on the present integration of histology with RT-qPCR, future work should expand the assessed molecular endpoints (e.g., aggrecan and COL1A1, inflammatory and catabolic mediators) and include protein-level and imaging biomarkers to strengthen mechanistic inferences [28,29,30]. Larger cohorts and dose-finding studies will be important to define optimal PRP concentrations and cell numbers, as well as to compare adipose versus bone marrow-derived MSCs under standardized conditions [28,29]. Ultimately, preclinical optimization should precede translational studies assessing safety, dosing, and delivery routes in clinically relevant settings.

The statistical analyses suggested that PRP infiltration—either alone or in combination with chondrocytes or MSCs—significantly improved the histological integrity of injured intervertebral discs compared with untreated injured discs. Although no statistically significant differences were observed between treated groups and healthy discs, the PRP-chondrocyte and PRP-MSC treatments improved scores toward healthy values, suggesting the regenerative benefits of these interventions. These findings support previous studies reporting the positive effects of PRP and stem cells in tissue regeneration, and align with the hypothesis that combining growth factors and cells may enhance IVD repair [29].

It is important to note that the variability in scores among the injured and treated discs suggests that individual factors may influence the response to treatment, underscoring the need for further research focused on optimizing therapies and personalizing the treatment modality for each case. Treatments with PRP—whether alone or in combination with chondrocytes or MSCs—appear to confer regenerative benefits in injured discs in the considered rabbit model. However, the observed variability indicates that complete IVD regeneration is complex and may require a more personalized approach.

## 4. Materials and Methods

### 4.1. Study Design

This study was a prospective, randomized, controlled experimental study involving an animal model.

### 4.2. Animal Model

Male New Zealand white rabbits with skeletal maturity corresponding to 4–5 months of age and weighing between 3 and 3.5 kg were used. The study was conducted in the animal facility of the Physiology Department at the Faculty of Medicine, Universidad Autónoma de Nuevo León (UANL). Based on the proposed study model and following the Institutional Animal Care and Use Committee (IACUC) recommendations for determining sample sizes in laboratory animal studies, a total of 15 rabbits were included [31]. These were divided into 3 groups of 5 rabbits:

- Group 1: PRP treatment;
- Group 2: PRP and chondrocytes;
- Group 3: PRP and mesenchymal stem cells (MSCs).

**Figure 8:**
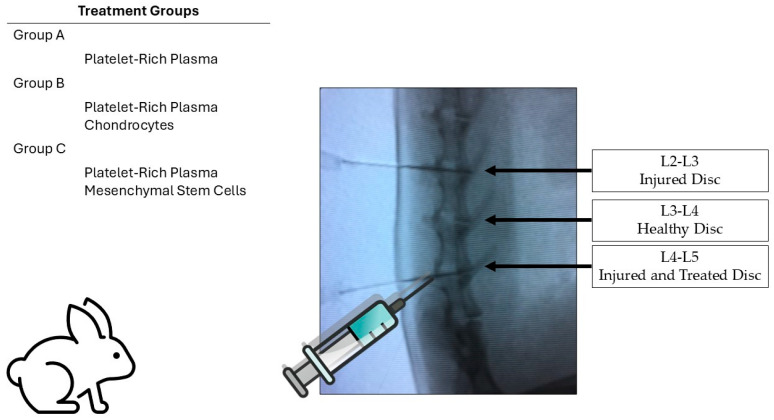
Experimental design and treatment groups. Rabbits were assigned to three experimental groups: Group A, Platelet-rich plasma (PRP); Group B, PRP combined with chondrocytes; and Group C, PRP combined with mesenchymal stem cells (MSCs). Each animal had three lumbar discs designated as follows: L2-L3, injured and left untreated (injured disc); L3-L4, healthy control disc; and L4-L5, injured and treated disc. The schematic image illustrates the injection procedure under fluoroscopic guidance, showing the anatomical locations of the treated and control discs.

The overall experimental design is illustrated in Figure 8, showing the distribution of treatment groups and disc levels used for injury, control, and treatment.

### 4.3. Study Groups and Experimental Design

Three groups of five rabbits were formed. Each rabbit had a positive control disc at the L2-L3 level, corresponding to the intervertebral disc injury site, while the L3-L4 disc served as an internal healthy control. The L4-L5 intervertebral disc was also injured and treated with PRP, PRP-MSCs, or PRP-chondrocytes, allowing for comparative assessment within each animal for the three discs (injured positive control, healthy internal control, and injured treated disc). The study groups were constituted as follows:

- PRP-treated group: Rabbits received PRP infiltration/treatment at eight weeks after the L4-L5 disc injury.
- MSC-treated group: Rabbits received infiltration/treatment with adipose-derived mesenchymal stem cells and PRP at eight weeks after the L4-L5 disc injury.
- Autologous chondrocyte-treated group: Rabbits received infiltration/treatment with autologous chondrocytes and PRP at eight weeks after the L4-L5 disc injury.

### 4.4. Induction of Intervertebral Disc Injury

The intervertebral disc degeneration model was established in 15 white New Zealand rabbits aged 4–5 months and weighing approximately 3–3.5 kg. Lumbar discs were punctured using a transparent table under fluoroscopic control.

In particular, the disc degeneration model was induced using an 18-gauge angiographic needle (Merit Advance^®^, South Jordan, UT, USA) via the percutaneous annular puncture technique developed by Masuda et al. [14], performed under fluoroscopic control. Unlike the original technique, no lumbotomy was performed, making it a fully percutaneous procedure.

Each rabbit was anesthetized via intramuscular injection of xylazine (5 mg/kg) and ketamine (35 mg/kg). The fur was shaved from the mid-back to the right side. The rabbits were placed in a prone position, with anteroposterior and lateral fluoroscopic views available. The L2-L3 and L4-L5 discs (previously injured for degeneration modeling) were identified via manual palpation of the interspinous space and confirmed via fluoroscopy.

An 18G angiographic needle (Merit Advance®, South Jordan, UT, USA) was inserted 3–4 cm ventral to the midline into the disc space under fluoroscopic control. Needle placement in the center of the disc was confirmed using anteroposterior and lateral fluoroscopic projections. Eight weeks after the initial puncture, degeneration was confirmed under fluoroscopic control via lateral X-ray projections, showing reduced disc height. The L3-L4 level remained unpunctured and served as a healthy internal control.

To minimize confounding effects, contact with the vertebral periosteum was minimized to avoid hypertrophy of the surrounding soft tissues and bone structures around the discs. Post-surgery, neurological symptoms were monitored in all rabbits. At 8 weeks after treatment, the animals were euthanized, and the L2-L3, L3-L4, and L4-L5 discs, along with the superior and inferior vertebral levels, were extracted in a single block.

### 4.5. PRP Preparation and Infiltration

Following aseptic techniques, 3 cc of whole blood was obtained from the auricular vein of the same donor rabbit. Blood was collected using insulin needles and transferred into vacutainer tubes containing 3.8% sodium citrate as an anticoagulant (BD Biosciences, San Jose, CA, USA). The tubes were gently inverted to ensure that a homogeneous mixture was obtained and centrifuged at 1314× *g* for 15 min.

After centrifugation, three layers were observed:

1. Erythrocytic layer (bottom);
2. Buffy coat (middle, containing leukocytes);
3. Plasma-rich and plasma-poor fraction (top, yellow layer).

The plasma-rich yellow layer was extracted with 1000 μL pipettes (Corning Inc., Corning, NY, USA), transferred into 15 mL Falcon tubes (Corning, NY, USA), and 250 μL of PRP was obtained. To this, 0.05 μL of calcium gluconate (10%) per ml of PRP was added and mixed, and the resulting solution was injected into the L4-L5 disc at four weeks after the initial puncture.

### 4.6. Chondrocyte Isolation, Culture, and Infiltration

Cartilage biopsies were obtained from the femoral condyles of the same donor rabbit. Using a lateral parapatellar approach, the patella was medially dislocated, and cartilage was harvested from the intercondylar groove using a surgical curette.

The biopsy was transported in PBS (Gibco, Thermo Fisher Scientific, Waltham, MA, USA) with gentamicin and amphotericin B (Gibco, Thermo Fisher Scientific, USA). Type II collagenase (Gibco, Thermo Fisher Scientific, USA) digestion (60 min), centrifugation (513× *g*, 10 min), and culture in OptiMem medium (Gibco, Thermo Fisher Scientific, USA) supplemented with 10% fetal bovine serum (FBS) (Gibco, Thermo Fisher Scientific, USA) and gentamicin (0.05 mg) for two weeks were performed. Cells (5 × 10^5^) were resuspended in 250 μL of PRP and injected into the L4-L5 disc at four weeks after injury.

### 4.7. MSC Isolation, Culture, and Infiltration

MSCs were obtained from adipose tissue (4–5 g, abdominal fat) and transported in PBS (Gibco, Thermo Fisher Scientific, USA) with antibiotics. The sample was washed, digested with 0.075% type I collagenase (Gibco, Thermo Fisher Scientific, USA) (30 min, 37 °C), centrifuged (329× *g*, 10 min), and resuspended in OptiMem I medium (Gibco, Thermo Fisher Scientific, USA) for two weeks. Cells (5 × 10^5^) were resuspended in 250 μL of PRP and injected at eight weeks after injury.

### 4.8. Treatment Administration and Follow-Up

At eight weeks after the induction of injury, intervertebral disc degeneration was confirmed via fluoroscopic evaluation based on a reduction in disc height. At this time point, treatments were administered under sterile conditions. A 1 cm midline skin incision followed by lumbotomy was performed to allow direct access to the L4-L5 disc space. Each rabbit received the previously assigned treatment:

- Group 1 received an intradiscal injection of PRP;
- Group 2 received PRP combined with chondrocytes;
- Group 3 received PRP combined with MSCs.

The L3-L4 disc remained uninjured throughout the experiment and served as the internal healthy control. After treatment, all animals were monitored and maintained for an additional 8-week period before euthanasia. Spinal segments encompassing L2-L3 (injured, untreated), L3-L4 (healthy control), and L4-L5 (injured and treated) were harvested en bloc for histological analysis.

### 4.9. Histological Processing and Analysis

The extracted discs were placed in glass jars with plastic screw caps, suspended in 4% paraformaldehyde solution (Sigma-Aldrich, St. Louis, MO, USA), decalcified, incubated at 37 °C for 15 days with continuous agitation, and embedded in paraffin. Histological sections (5 μm) were obtained from the center of the paraffin blocks, placed on slides, and stained with hematoxylin and eosin (H&E) (Sigma-Aldrich, USA) for histological analysis. Slides were examined at 4×, 10×, and 40× magnifications.

### 4.10. Histological Scoring and Evaluation Criteria

The extracted intervertebral disc blocks were decalcified separately and incubated at 37 °C with agitation for 15 days. Subsequently, 3 mm transverse sections were obtained from each sample and embedded in paraffin. Histological sections of 5 µm were cut from the center of the paraffin blocks, mounted on slides, and stained with hematoxylin and eosin for microscopic evaluation at 4×, 10×, and 40× magnifications. The histological assessment followed the grading scale established by Masuda et al. [14], which categorizes disc degeneration into four parameters and enables scores from 4 (normal disc) to 12 (severely degenerated disc) to be assigned (Table 2). This grading system was applied to evaluate the degeneration severity in the injured L2-L3 discs, the integrity of the healthy control L3-L4 discs, and the regenerative response in the injured and treated L4-L5 discs across the three treatment groups (PRP, PRP-MSCs, PRP-chondrocytes). For histological data, parametric variables were analyzed via Student’s t-test and non-parametric variables via chi-square test using IBM SPSS Statistics for Windows, version 25.0 (IBM Corp., Armonk, NY, USA).

### 4.11. RNA Extraction from FFPE Blocks

Five-micrometer sections were cut from formalin-fixed, paraffin-embedded tissue blocks and deparaffinized in xylene, followed by a graded ethanol series. Total RNA was isolated using the RecoverAll™ Total Nucleic Acid Isolation Kit for FFPE (Thermo Fisher Scientific; Cat. No. AM1975), according to the manufacturer’s instructions, yielding approximately 1–2 µg RNA per sample.

### 4.12. cDNA Synthesis

An aliquot of 500 ng RNA was treated with DNase I (included in the RecoverAll™ kit) and reverse-transcribed in a 20 µL reaction using the High-Capacity cDNA Reverse Transcription Kit (Thermo Fisher Scientific) under the following conditions: 10 min at 25 °C, 120 min at 37 °C, and 5 min at 85 °C.

### 4.13. Quantitative Real-Time PCR

Reactions were run on a QuantStudio 5 Real-Time PCR System (Thermo Fisher Scientific) using PowerUp™ SYBR™ Green Master Mix (Thermo Fisher Scientific). Each 20 µL reaction mixture contained 10 µL of Master Mix, 0.5 µM of each primer, and 2 µL of a 1:10 dilution of cDNA. Thermal cycling parameters were 95 °C for 2 min; 40 cycles of 95 °C for 15 s and 60 °C for 60 s; followed by a melt-curve analysis from 60 °C to 95 °C. Primers were adopted from Huang et al. [32]: for COL2A1, the forward primer was 5′-GGGTCCTTTGGCTGTTCAGA-3′ and the reverse primer was 5′-TTCTCCCCAGAAACACACCG-3′; for COL10A1, the forward primer was 5′-GGCTTCCCAGTGGCTGATAG-3′ and the reverse primer was 5′-TTTTGCTCTCTCTGGGTGGC-3′. β-actin was used as the endogenous control, with forward primer 5′-AGACCACCTTCAACTCGATCAT-3′ and reverse primer 5′-ACTCGTCATACTCCTGCTTGCT-3′. Each sample was run in technical triplicate, and specificity was confirmed through a single peak in the melt-curve and absence of primer dimers. Cycle threshold (Ct) values were obtained using the QuantStudio 5 software. Relative expression was calculated using the 2^−ΔΔCt^ method, normalized to β-actin, and using the lesion control (L2-L3) from each animal for calibration. Data are presented as the mean ± SD (n = 5). Statistical analyses were performed with IBM SPSS Statistics for Windows, version 25.0 (IBM Corp., Armonk, NY, USA), including one-way ANOVA and Tukey’s post hoc test (*p* < 0.05).

## 5. Conclusions

PRP treatments—both alone and in combination with chondrocytes or MSCs—not only improved histological scores but also induced a clear molecular shift toward a more regenerative phenotype, as evidenced by up-regulation of the chondrogenic marker COL2A1 and down-regulation of the hypertrophic marker COL10A1. Notably, the PRP-chondrocytes protocol produced the most pronounced improvements at both the tissue and gene expression levels, whereas PRP-MSCs yielded intermediate benefits and PRP alone elicited more modest changes. However, even the strongest responses fell short of fully restoring healthy disc characteristics, underscoring that intervertebral disc regeneration remains a complex, multifactorial challenge likely requiring finely tuned combinations of cellular, molecular, and delivery parameters.

This study highlights the potential of regenerative therapies in intervertebral disc repair, which could significantly contribute to future treatment strategies for discogenic diseases. Continued research aimed at optimizing PRP formulations, refining the properties of MSCs and chondrocytes, and standardizing delivery protocols will be essential to maximize therapeutic outcomes and ensure reproducible clinical translation.

Although this study has certain limitations, it also defines valuable opportunities for further research. The relatively small sample size reflects the exploratory nature of this preclinical work but still yielded consistent histological and molecular trends. Likewise, the absence of groups treated with chondrocytes or MSCs alone limited direct comparisons regarding their isolated effects yet allowed us to focus on the synergistic behavior of cell–PRP combinations, which was the central hypothesis of this study. Finally, while the present results were obtained in a short-term rabbit model, they provide a strong biological foundation for subsequent translational studies. Expanding the duration, sample size, and inclusion of cell-only controls in future experiments are expected to help in validating and extending these encouraging findings toward clinical application.

## Figures and Tables

**Table 1:** Scores obtained among the three treatment groups.

		Score			
		Healthy Disc n = 15	Injured Disc n = 15	Injured and Treated Disc n = 15	^Ţ^ P
Group	PRP	4.4 ± 0.54	10 ± 1.5	7.2 ± 0.83	<0.001
	PRP-chondrocytes	4.8 ± 0.83	10.8 ± 0.44	6.4 ± 0.54	<0.001
	PRP-MSCs	5 ± 0.7	10.8 ± 1.09	7.2 ± 0.44	<0.001
	^Ŧ^ P	0.42	0.46	0.11	

Each superscript indicates differences between subgroups. ^Ţ^ One-way ANOVA comparing the scores obtained among the different disc types evaluated. ^Ŧ^ One-way ANOVA comparing the scores obtained among the treatment groups.

**Table 2:** Histological grading scale *.

I. Annulus Fibrosus Grade:
Normal fibrocartilage lamellae pattern (U-shaped posteriorly and slightly convex anteriorly) with no broken or wavy fibers in any part of the annulus. Broken or wavy fibers in less than 30% of the annulus. Broken or wavy fibers in more than 30% of the annulus.
II. Boundary Between Annulus Fibrosus and Nucleus Pulposus:
Normal. Minimally disrupted. Moderately/severely disrupted.
III. Nucleus Pulposus Cellularity:
Normal cellularity with large vacuoles in the gelatinous matrix structure. Slight reduction in cell number and fewer vacuoles. Moderate/severe (50%) reduction in cell number with no vacuoles.
IV. Nucleus Pulposus Matrix:
Normal gelatinous appearance. Slight condensation of the extracellular matrix. Moderate/severe condensation of the extracellular matrix.

* Based on four categories of degenerative changes, with scores ranging from a normal disc with 4 points (1 point per category) to a severely degenerated disc with 12 points (3 points per category).

## Data Availability

The data presented in this study are all available in this article.
