# Correction: Reyes-Fernández et al. Comparative Regenerative Efficacy of PRP Combined with Chondrocytes or Mesenchymal Stem Cells for Intervertebral Disc Regeneration in a Rabbit Model. *Int. J. Mol. Sci.* 2025, *26*, 10007

**DOI:** 10.3390/ijms27052329

**Published:** 2026-03-02

**Authors:** Pedro M. Reyes-Fernandez, Viktor J. Romero-Díaz, Jaime García Juárez, José F. Vílchez-Cavazos, Rodrigo Elizondo-Omaña, Carlos A. Acosta-Olivo, Víctor M. Peña-Martínez, Jorge Lara-Arias

**Affiliations:** 1 Orthopedics and Traumatology Service, “Dr. José Eleuterio González” University Hospital, Universidad Autónoma de Nuevo León, Monterrey 66460, Mexico; 2 Histology Department, Faculty of Medicine, Universidad Autónoma de Nuevo León, Monterrey 66460, Mexico; 3 Anatomy Department, Faculty of Medicine, Universidad Autónoma de Nuevo León, Monterrey 66460, Mexico

## Addition of an Author

Rodrigo Elizondo-Omaña was not included as an author in the original publication [1] due to an internal oversight during manuscript preparation and online submission. The in vivo rabbit work (model establishment and facilitation of animal housing/surgical conditions) was completed approximately 18 months prior to submission with support from the Anatomy Department, whereas later stages (histology processing/analysis and manuscript integration/revisions) were performed across different units and coordinated through the University Hospital team. During drafting and submission, the author list relied on an internal working version and Dr. Elizondo-Omaña was inadvertently not carried over into the submission system; unfortunately, this was not detected during the revision rounds. Dr. Elizondo-Omaña made substantial intellectual and practical contributions to the in vivo component of the study. Specifically, he established and implemented the rabbit experimental model, ensured the institutional and operational conditions for animal housing and welfare, and facilitated the infrastructure and operative setting required for rabbit maintenance and surgical procedures. He also contributed to the methodological development and oversight of the animal procedures and reviewed the manuscript sections relevant to the animal model and in vivo methods. The additional affiliation and corrected Author Contributions statement appears below. The authors state that the scientific conclusions are unaffected. This correction was approved by the Academic Editor. The original publication has also been updated.

^3^  Anatomy Department, Faculty of Medicine, Universidad Autónoma de Nuevo León, Monterrey 66460, Mexico

**Author Contributions:** Conceptualization, J.L.-A. and V.M.P.-M.; methodology, P.M.R.-F., R.E.-O., V.J.R.-D. and J.G.J.; investigation, P.M.R.-F., R.E.-O., V.J.R.-D. and J.G.J.; resources, J.F.V.-C., C.A.A.-O. and R.E.-O.; data curation, P.M.R.-F., V.J.R.-D. and J.G.J.; formal analysis, P.M.R.-F., V.J.R.-D. and J.G.J.; writing—original draft preparation, P.M.R.-F.; writing—review and editing, P.M.R.-F., V.J.R.-D., J.G.J., J.F.V.-C., R.E.-O., C.A.A.-O., V.M.P.-M. and J.L.-A.; supervision, J.L.-A., V.M.P.-M., J.F.V.-C. and C.A.A.-O.; project administration, J.L.-A. and V.M.P.-M. All authors have read and agreed to the published version of the manuscript.
